# Methane and Carbon Dioxide Hydrate Formation in the Presence of Metal-Based Fluid

**DOI:** 10.3390/ma15238670

**Published:** 2022-12-05

**Authors:** Omar Nashed, Behzad Partoon, Bhajan Lal, Khalik Mohamad Sabil, Sana Yaqub, Azmi Mohd Shariff

**Affiliations:** 1 Department of Chemical Engineering, Faculty of Technical Engineering, Bright Star University, El-Brega 218645, Libya; 2 Department of Biological and Chemical Engineering, Aarhus University, Universitetsbyen 36, 8000 Aarhus, Denmark; 3 Chemical Engineering Department, Universiti Teknologi PETRONAS, Bandar Seri Iskandar 32610, Perak, Malaysia; 4 CO_2_ Research Centre, Universiti Teknologi PETRONAS, Bandar Seri Iskandar 31750, Perak, Malaysia; 5 PETRONAS Research Sdn Bhd, Kawasan Institusi Bangi, Lot 3288 3289 Off Jalan Ayer Itam, Kajang 43000, Selangor, Malaysia; 6 U.S. Pakistan Centre for Advance Studies in Energy, Department of Thermal Energy Engineering, National University of Science and Technology, Islamabad 44000, Pakistan

**Keywords:** gas hydrates, kinetic hydrate promoters, solid particles, rate constant, hydrate kinetics, nanomaterial

## Abstract

Hydrate-based technology has yet to find its way to commercial applications due to several issues, including formation conditions and slow kinetics. Several solid particles were introduced to speed up hydrate formation. However, these solid compounds have given contradictory results. This study investigated the effect of high thermal conductive metallic nanofluids of silver (Ag) and copper (Cu) on CH_4_ and CO_2_ hydrates. The solid particles were suspended in a 0.03 wt% SDS aqueous solution, and the results were compared with the 0.03 wt% SDS and deionized water samples. A stirred tank batch reactor was used to conduct the thermodynamic and kinetic experiments. The thermodynamic study revealed that 0.1 wt% of solid particles do not shift the equilibrium curve significantly. The kinetic evaluation, including induction time, the initial rate of gas consumption, half-completion time, *t*_50_ and semi-completion time, *t*_95_, gas uptake, and storage capacity, have been studied. The results show that the Ag and Cu promote CH_4_ hydrates while they inhibit or do not significantly influence the CO_2_ hydrates formation. A predictive correlation was introduced to get the apparent rate constant of hydrate formation in the presence of metal-based fluid at the concentrations range of 0.005–0.1 wt%.

## 1. Introduction

Clathrate hydrates (gas hydrates) are crystalline inclusion compounds where a small guest molecule is encapsulated inside water cavities under appropriate thermodynamic conditions [1]. Gas hydrates have been renowned as a severe issue for the oil and gas industry due to the blockage caused by hydrate formation in the pipelines [2]. However, gas hydrates have shown a bright side for several gas applications such as separation, transportation, and storage [3,4,5]. Many unique advantages inspired scientists to investigate the potential applications of gas hydrates. Up to 170 m^3^ of gas under standard conditions can be stored in 1 m^3^ of gas hydrates [6]. Moreover, gas hydrates can treat multi-component gas mixtures and consider green technology as no or deficient concentrations of chemical additives are required. Not to forget its non-explosive nature and low cost of hydrate-based gas transportation compared with liquefied gas [7]. Additionally, guest gas is kept in its respective molecular form, and relatively full gas recovery or utilization is achievable. On the other hand, there are several challenges to the industrial development of hydrate-based technology. Hydrate formation is a slow stochastic crystallization process in nature, depending on the system condition, and could have very slow kinetics. The hydrate formation started with the development of nuclei in the solution, also known as induction time. When the size and number of these nuclei reach a critical value, massive crystal growths will start. During this time, fast gas consumption will be observed, and considerable heat of hydrate formation will release to the liquid phase. During the crystal growth, an excellent gas/liquid/solid interface is required to fill all cavities in the structure, and adequate heat evacuation is required to keep the favorable hydrate condition [8]. When the retention time is short, especially during continuous production of gas hydrate, inadequate gas occupancy is widespread [9].

To overcome these issues, researchers have looked at several chemical additives [10]. These additivities either act as thermodynamic promoters or kinetics ones. The thermodynamic promoters, such as tetrahydrofuran, improve the hydrate formation process. However, they decrease the storage capacity of gas hydrates as they occupy a portion of cages in the crystalline structure of gas hydrate. On the other hand, kinetics promoters, such as surfactants, only enhance the kinetics. According to several studies, sodium dodecyl sulfate (SDS) is the most promising kinetic hydrate promoter among different types of surfactants [6,10]. It accelerates the hydrate formation by lowering the surface tension at the water/gas boundary and consequently enhances gas uptake [6,10]. However, they have disadvantages that limit their applications. The promotion impact of surfactants is concentration-dependent, and a minimum concentration is required to show adequate promotion effect [10]. However, at the hydrate formation conditions, i.e., high pressures and low temperatures, many surfactants have poor solubilities, which could cause the surfactants to precipitate before an effective concentration is attained [11]. Furthermore, the presence of surfactant, especially at higher concentrations, can lead to the foaming problem during the formation and dissociation of gas hydrate. Foaming problems would also be a challenge during the hydrate dissociation. This would cause an outflow of the liquid solutions during gas hydrate processing. In addition, hydrate growth occurs along the reactor wall when surfactants are used due to the capillary effect [12]. Although this effect can increase the formation rate, this may limit the utilization area of the reactor, and additional separation and compaction of the hydrates are impractical [11]. As nanofluids can increase heat and mass transfer, they were proposed and investigated as gas hydrate promoters. They have advantageous properties such as large surface area, high thermal conductivity, recyclability, eco-friendly, and reasonable cost.

The term “nanofluid” describes a collision of nanoparticles, mostly below 100 nm, dispersed in a liquid phase. Several dispersion techniques are used to prepare the nanofluids, such as surface modification of nanoparticles, adding surfactants to the base liquid, and ultrasonication [13]. In 2006, copper nanoparticles were utilized by Li et al., as a hydrate promoter for 1,1,1,2-Tetrafluoroethane (HFC134a) [14]. The inherent high thermal conductivity of copper was the main reason for choosing it to evacuate the heat of exothermic hydrate formation from the reactor. After that, several studies explored different kinds of nanomaterials, including silver [15,16,17], copper oxide [18,19], zinc oxide [20,21,22], and single and multi-wall carbon nanotubes [7,23]. The metals have attracted more attention due to their high thermal conductivity, specifically silver and copper. As previously stated, Cu nanoparticles are the first nanomaterial investigated for potential application in gas hydrate production by Li and his team [14]. They investigated how HFC134a, a refrigerant for air-conditioning systems, forms gas hydrate and dissociates in the presence of Cu at concentrations of 0.1 and 1 wt%. Nano-Cu was mixed with 0.04 wt% sodium dodecylbenzenesulfonate-6 as a dispersant agent. They observed that nano-Cu reduced the induction time. This effect was enhanced by increasing Cu loading. Authors attributed the enhancement to the improved heat transfer, the availability of nucleation sites, the large specific surface area, and enhanced mass transfer [14]. After that, Arjang and his co-workers used silver particles to promote CH_4_ hydrate formation [24]. According to Arjang et al., the observed promotion effect was due to a greater nanofluid heat transfer, significant surface-to-volume ratio boost, and heterogeneous nucleation caused by solid particles.

Yang studied 0.15 wt% copper particles (CP) with a size range of 3–10 μm suspended in 0.05 wt% SDS. The mixture was blended with stainless steel fibers (SSF) with an average diameter of 8 μm. The results showed that the metallic additives accelerated hydrates nucleation and enhanced heat transfer. In addition, it needed less time to attain the storage capacity of 100 V/V by using the SDS/CP–SSF suspension compared to the SDS/CP system and the simple SDS solution [25].

In another study, sole Cu nanoparticles and Cu dispersed in a cationic surfactant cetyltrimethylammonium bromide (CTAB) solution were studied [19]. The reported induction times revealed that the 2.2 × 10^−3^ M CTAB sample had the shortest methane hydrate induction time (at 5.5 MPa and 275.15 K). Sole copper particles with higher concentrations of 1.57 × 10^−2^ M and 1.57 × 10^−1^ M appeared more effective as they lowered the average induction time compared to Cu/CTAB nanofluids. On the contrary, an inverse proportion is found between Cu/CTAB nanofluids concentration and the induction time. The results showed the system’s increased inhomogeneity, promoting heterogeneous nucleation. Moreover, among the various compositions of nanofluid, methane hydrate production in water-based copper nanofluids with comparatively high concentrations of 1.57 × 10^−2^ M and 1.57 × 10^−1^ M has the highest apparent rate constant [19].

Silver nanoparticles were investigated as potential hydrates promoters for carbon dioxide, methane, and ethane [16,24,26,27]. A study showed that silver could shorten the induction time of CH_4_ hydrate by 85% and 73.9% at 4.7 MPa and 5.7 MPa, respectively [24]. Another study revealed that Ag and their mixture with SDS did not significantly influence the induction time due to rapid CO_2_ hydrate formation in pure water, i.e., about 1 min. [28]. Recent work reported a promotional impact of grafted silver nanoparticles (2–5 nm) on the SDS-coated nanospheres (Ag&SDS@PSNS)(about 100 nm) [29]. In another work, Ag was immobilized on activated carbon to improve the performance of activated carbon as a kinetic promoter [30]. However, another investigation showed that the SDS or silver nanoparticles did not significantly affect CO_2_ consumption within 0–120 min [28]. Cu and Ag also considerably influenced the amount consumed by the gas mixture (75 mol% CH_4_ and 25 mol% CO_2_) at the initial stage of the experiment. However, it returned to a negative one at the end of the experiment [31].

From the above literature revision, it can be concluded that no consensus has yet to be reached on the effect of metallic particles on gas hydrate formation kinetics. Moreover, it is unclear which type of particles must be chosen for a specific gas. As hydrate kinetic data are stochastic and cannot be compared with each other work unless identical conditions, equipment, and procedures are used, this work investigates the impact of copper and silver on methane and CO_2_ hydrate formation. Pure water and SDS solution were used as references to evaluate the performance of Ag and Cu particles. The impact of Cu and Ag on the thermodynamics and kinetics of CO_2_ and CH_4_ hydrate formation has been studied experimentally. The hydrate equilibrium temperature, induction time, initial rate of gas consumption, and storage capacity were measured during the hydrate formation. Moreover, the rate constant was calculated and correlated with the concentration.

## 2. Materials and Methods

### 2.1. Material

Carbon dioxide and methane were obtained from Air Product Sdn. Bhd. (Perak, Malaysia). Silver and copper were purchased from Research Nanomaterials Inc. (Houston TX, USA). The nanofluids were prepared in 0.03 wt% sodium dodecyl sulfate (SDS) aqueous solution, which was supplied by Merck (Rahway, NJ, USA). Deionized water (The resistivity is 18 MΩ-cm) was used to prepare all nanofluids. The chemical name, purity, supplier, and size of the metal powder are listed in Table 1. The concentration of nanoparticles is in the range of 0.01–0.1 wt%. SDS was used as a stabilizer and reference to evaluate the results. An electronic balance with an accuracy of ±0.0001 g was used to prepare the samples.

### 2.2. Apparatus

**Figure 1:**
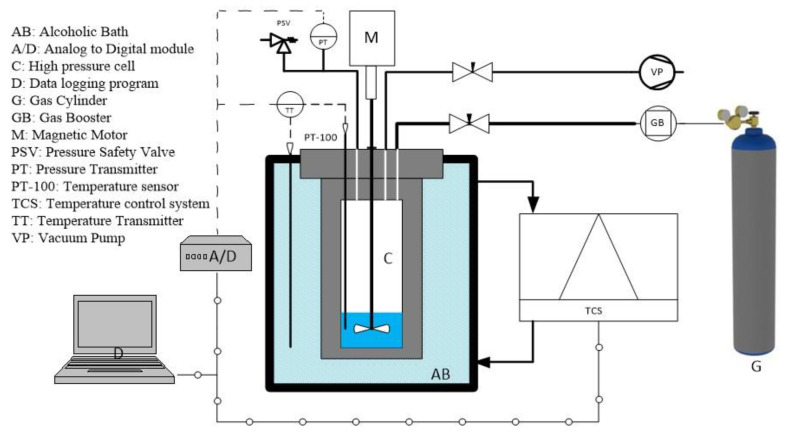
Experimental setup used in this study.

Figure 1 depicts the experimental setup employed in this study. A 423 mL stainless-steel cell was used to form hydrate. The vessel is designed to work at a maximum pressure of 20.0 MPa. The pressure was measured by a pressure transducer connected to the cell. Two thermocouples were used to measure the temperatures in the gas and liquid phases with an accuracy of ±0.5 K. A 2-bladed pitch impeller with magnetic coupling to a DC motor generated stirring up to 600 rpm was used to mix the various phases. To maintain the temperature at the desired level, the vessel was placed in a thermostatic glycol bath. More details on the equipment specification are described in our previous works [7,23].

### 2.3. Procedure

First, 100 mL of the liquid sample was placed into the cell. Once closed, the vacuum pump was used to remove the air from the cell. Then, the gas used in the experiment, either CH_4_ or CO_2_, was injected three times to purge the vessel and ensure complete air removal. As thermodynamic and kinetic experiments have different experimental procedures, the following sections explain the steps and conditions of each.

#### 2.3.1. Hydrate Equilibrium Temperature Measurement

As a standard phase equilibria practice, the equilibrium point for gas hydrate measurement is the dissociation hydrate temperature, commonly measured via the T-cycle method [20]. This method was followed by decreasing the vessel temperature to 273.15 K. Sufficient time was given at constant temperature to ensure complete hydrate formation indicated by stable pressure. The temperature was then raised using the stepwise heating approach, with an increment of 0.25 K every 1 h. The reliable measurement of the hydrate dissociation temperature required a slow heating rate. To get the equilibrium curve, four different pressures between 3.1 and 8.2 MPa for CH_4_ and 1.7 to 4.6 MPa for CO_2_ were used in the experiments.

#### 2.3.2. Evaluation of Kinetics of Hydrate Formation

For the kinetic testing, the cell was cooled down to 281.65 K, corresponding to a temperature 2 K above the equilibrium temperature of CH_4_ and CO_2_ hydrates at 5.1 MPa and 2.7 MPa, respectively. Then, the gas was injected into the cell up to desired pressure. Then, the system was allowed to approach equilibrium after the stirrer was turned on. After reaching constant pressure and temperature, the cell was cooled down to 274.15 K without stirring during the cooling process. As the temperature decreased, a decrease in pressure was observed. When the temperature became constant at 274.15 K and the pressure stabilized after 65 min, the stirrer was turned on. The data acquisition system recorded the pressure and temperature every 10 s. Once a sudden simultaneous decrease in vessel pressure and an increase in temperature is observed, hydrate formation is inferred. The experiment then kept running until the pressure stabilized for 2 to 3 h, indicating that the hydrate formation was completed.

Several indexes were measured and calculated to assess the effect of metal-based fluids on the kinetic of hydrate formation. The time required to produce a detectable hydrate nucleus is called induction time [8]. It is determined by observing a sudden decrease in pressure associated with a sharp rise in temperature and gas consumption. The stochastic nature of induction time poses the need to repeat the experiments. Therefore, the experiments have been repeated three times at least.

Next, the initial gas consumption rate was calculated as it is a crucial parameter for hydrate-based applications. It can be calculated according to Equation (1) as follow:

(1) $$r\left(t\right)=-\frac{n_{i}^{i-1}-n_{i}^{i+1}}{t_{i-1}-t_{i+1}}n_{w_{0}}^{-1}$$

where $n_{i}^{i-1}$ and $n_{i}^{i+1}$ are the mole numbers of gas in the gas phase at time intervals $t_{i-1}$ and $t_{i+1}$, respectively, and $n_{w_{0}}$ is the initial mole number of the water.

An essential factor in advancing gas hydrate technology to the industrial scale is the quantity of gas required to carry out the maximal hydrate formation. Equation (2) is used to calculate the gas consumption. It is assumed that the volume of the water does not change when the hydrate forms. Therefore, the isothermal experiment employs the following equation:

(2) $$\Delta n_{g}=\frac{V}{R}\left[{\left(\frac{P}{ZT}\right)}_{0}-{\left(\frac{P}{ZT}\right)}_{t}\right]$$

where *T* and *P* denote the system temperature and pressure, respectively. *R* is the universal gas constant, *V* is the gas’s volume, and *Z* denotes the compressibility factor, which is determined using the Peng-Robinson Equation of State. Subscript 0 stands for the start time of the experiment, and *t* represents the conditions at time *t*. The amount of gas consumption was divided by the number of moles of water to get the gas uptake as shown in Equation (3). Kinetics in gas hydrate is equipment-dependent, and gas uptake, a normalized value, makes the results independent of sample size and is a better parameter for comparing the results with others.

(3) $$U=\frac{\Delta n_{g}}{n_{w}}$$

Furthermore, it is vital to know the storage capacity (SC) before storing the gas in hydrate form. The storage capacity refers to the ratio of the volume of captured gas under standard conditions (STP) to the volume of hydrate and is calculated using the following Equation (4):

(4) $$SC=\frac{V_{{}^{g}}^{STP}}{V_{H}}=\frac{\Delta n_{g}RT^{STP}/P^{STP}}{V_{H}}$$

where $V_{H}$ is the gas hydrate volume calculated using Equation (5) [24].

(5) $$V_{H}=M\times \Delta n_{g}\times v_{W}^{\beta }$$

where $v_{W}^{\beta }$ is the molar volume of the empty hydrate lattice, calculated following Equation (6):

(6) $$\nu_{w}^{\beta }={\left(11.835+2.217\times 10^{-5}T+2.242\times 10^{-6}T^{2}\right)}^{3}\times \frac{10^{-30}N_{A}}{46}-8.006\times 10^{-9}P+5.448\times 10^{-12}P^{2}$$

where *N_A_* is the Avogadro number. The physical reaction for studied gas hydrate can be stated as the following (Equation (7)):

(7) $$\mathrm{Gas}+MH_{2}O↔\mathrm{Gas}.{\left(H_{2}O\right)}_{M}$$

where *M* represents the hydration number and is defined as the number of water molecules per guest molecule. The fractional occupancy of the small and large cavities is directly correlated with the hydration number as follows (Equation (8)):

(8) $$M=\frac{46}{6\theta_{l}+2\theta_{s}}$$

where *θ_l_* and *θ_s_* are the fractional occupancy of large and small cavities, respectively. The Langmuir adsorption theory is used to calculate the fractional occupancy as follows (Equation (9)) [32]:

(9) $$\theta_{i}=\frac{C_{i}f_{g}}{1+C_{i}f_{g}}$$

where *C_i_* is the gas Langmuir constant of gas in a type *i* cavity and $f_{g}$ is the fugacity of the gas in the gas phase. The Langmuir constant of gas ($C_{i}$) is expressed according to Equation (10):

(10) $$C_{i}=\frac{A_{i}}{T}\exp\left(\frac{B_{i}}{T}\right)$$

where *A_i_* and *B_i_* are constants and *T* is the temperature in Kelvin. Peng-Robinson equation of state is used to get the fugacity of gases in the gas phase.

#### 2.3.3. Rate Constant Modeling

The kinetic model used in this study was proposed in 1987 by Englezos et al. [33]. The driving force in this model is defined as the fugacity difference between the dissolved gas and the hydrate three-phase equilibrium at the experimental T. A Mass transfer-based model was developed based on the crystallization growth theory for hydrate particles and the two-film theory. Unlike chemical reaction models, this model is significantly less dependent on T. The hydrate particle growth, according to the Englezos model, occurs in two steps as follows:

(a) Diffusion of the gas from the bulk of the solution to the hydrate-liquid interface.

(b) Adsorption of the gas molecules to be incorporated into the water cages and stabilize the water cages.

The model assumed a spherical and uniform distribution of the hydrate nucleus in the liquid phase. With the overall driving force defined earlier, the growth rate per particle was expressed by Equations (11) and (12).

(11) $$r=\left(\frac{dn}{dt}\right)=K^{*}A_{p}\left(f-f_{eq}\right)$$

where K^*^ is the apparent rate constant that represents the combination of the rate constant for the diffusion and adsorption processes. *A_p_* is each particle’s surface area. *f* is the fugacity of the gas, and *f_eq_* is the fugacity of the gas at equilibrium.

(12) $$\frac{1}{K^{*}}=\frac{1}{k_{r}}+\frac{1}{k_{d}}$$

In Equation (12), *k_r_* is the intrinsic rate constant for the hydrate particle growth reaction, and *k_d_* is the mass transfer coefficient around the particle. As the experiments are conducted in stirred tank reactor, mass transfer resistances around the particle are excluded, then *k_d_* >> *k_r_* and K^*^ ≈ *k_r_*. Therefore, several researchers amended the Englezos model and used it in the following form, Equation (13):

(13) $$r=\left(\frac{dn}{dt}\right)=K_{app}\left(f_{g}-f_{eq}\right)$$

The apparent rate constant *K_app_* values were calculated at each data point for pure CO_2_ and pure CH_4_ hydrates using the initial gas consumption rate. The average *K_app_* was taken and reported as *K_exp_*. The average value can be reported for every concentration and used to recalculate the initial gas consumption rate. However, this method leads to an overfitted estimation that only makes the results applicable to the specified system. Therefore, the correlation between the concentration and the rate constant for each chemical was found to calculate the rate constant as a function of the concentration. The first stage in the modeling process is illustrating the data graphically and identifying the trends. The scatter plot of the apparent rate constant *K_app_* versus concentration was depicted. The best curve to describe the correlation between the two variables was estimated using the least square regression. The correlations were validated by predicting *K_app_* at tested concentrations.

The chosen model was used to predict each concentration’s initial gas consumption rate. The mean absolute percentage error *MAPE*% is calculated to evaluate the accuracy of the prediction model using Equation (14):

(14) $$MAPE\%=\frac{100}{N}\sum \frac{K_{pre}-K_{exp}}{K_{exp}}$$

where *K_exp_* and *K_pre_* are the experimental and predicted rate constants.

## 3. Results and Discussion

### 3.1. Characterization of the Silver and Copper Particles

**Figure 2:**
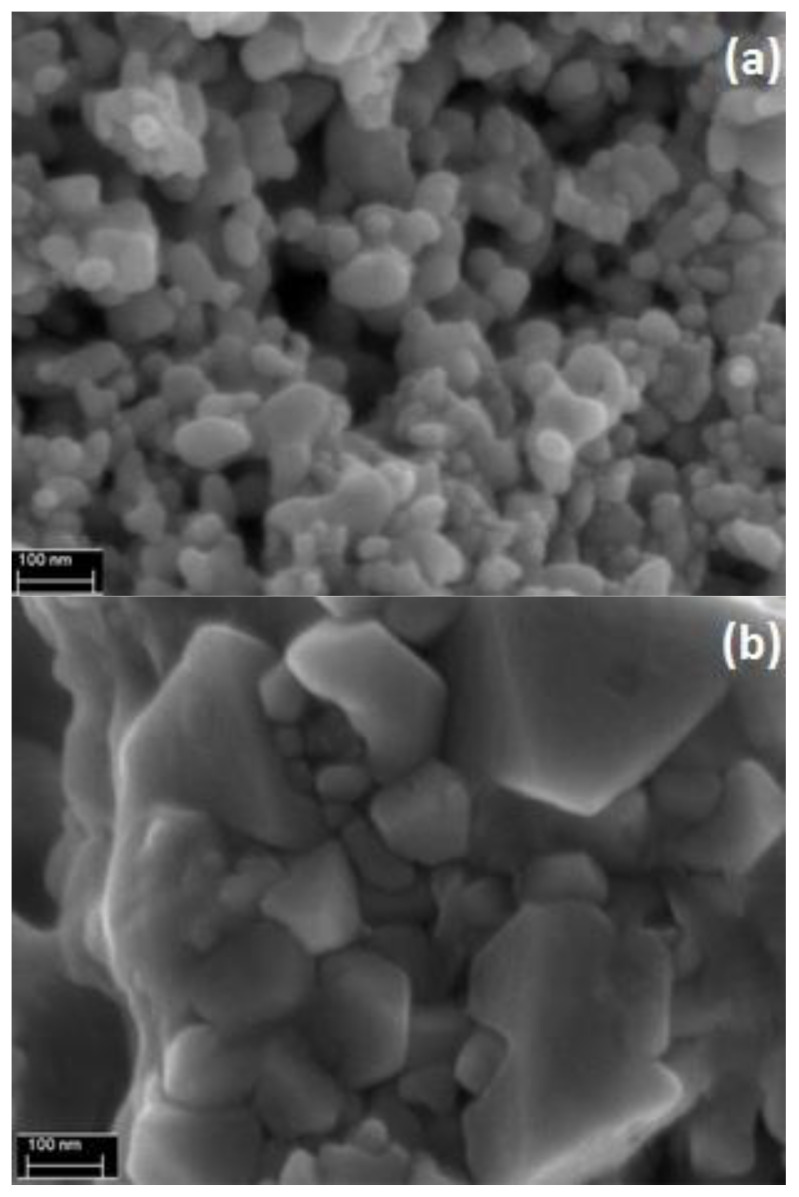
FESEM image at magnitude ×100,000 and scale of 100 nm for (**a**) Ag and (**b**) Cu.

The Field Emission Scanning Electron Microscope (FESEM) images of Ag and Cu are displayed in Figure 2. In both images, amorphous morphology was observed as no precise shape was detected. It also can be observed that Ag particles are in nano size as they are in dimensions less than 100 nm. On the other hand, Cu particles are in micro size. In addition, the surface area was measured by the N_2_ adsorption technique and evaluated by the BET equation. The BET measurements were conducted by *ASAP 2020* from *micromeritics*. The test chamber was under cryogenic conditions (−196 °C), and nitrogen was used to measure the surface area. Surprisingly, Cu particles showed a larger surface area though they have a more prominent size than Ag. The BET surface areas are 2.39 m^2^/g and 3.36 m^2^/g for Ag and Cu, respectively. To extend the characterization of the solid particles, the EDX test was conducted along with FESEM. The sample of Ag is found as pure silver, while a significant amount of oxygen atoms was detected in the Cu sample. The copper particles slowly react with the atmospheric oxygen to form a layer of copper oxide. Therefore, surface area, measured by the N_2_ adsorption method, is not governed by the size of particles only, but it depends on surface functionalities, number of active sites, and accessible surface area/pores on the adsorbent. If particles are smaller but do not have sufficient charged sites and accessible pores can result in lower adsorption capacity.

### 3.2. Hydrate Phase Equilibrium in the Presence of Silver and Copper Particles

**Figure 3:**
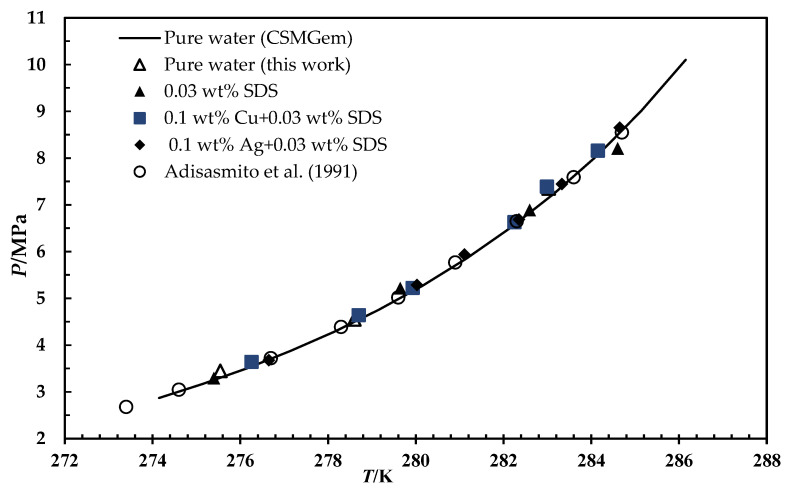
HLVE curve of methane for pure water (CSMGem software, Adisasmito et al. [35] and this work), 0.03 wt% SDS, and 0.1 wt% metal-based fluids.

**Figure 4:**
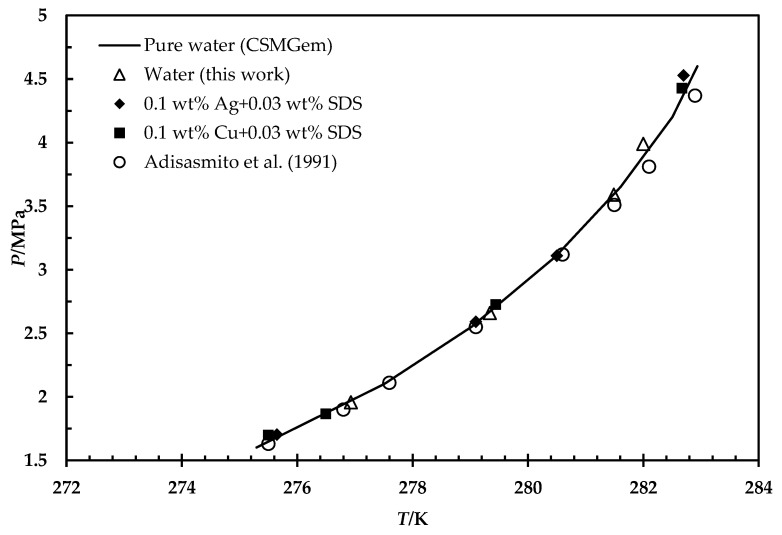
HLVE curve of CO_2_ hydrate for pure water (CSMGem software, Adisasmito et al. [35] and this work), 0.03 wt% SDS, and 0.1 wt% metal-based fluids.

The system’s phase boundary data are necessary to process the gases for any application. To study the impact of metallic particles on CH_4_ and CO_2_ hydrate phase equilibrium, thermodynamic tests have been carried out using 0.1 wt% of metallic particles dispersed in an aqueous solution containing 0.03 wt% SDS. To confirm the accuracy of the equipment and procedure, the experiments were first carried out using a blank sample, and the results were compared with those from the CSMGem software. The outcomes are consistent with the predicted data, as seen in Figure 3. Then, the 0.03 wt% SDS and metal-based fluids samples were examined. It is worth mentioning that the majority of researchers believe that surfactants are only kinetics promoters, and they will not change the phase boundaries of the system [6,34]. Similarly, the findings reveal an insignificant effect on the hydrate phase equilibria for all the tested SDS and metal particles, as illustrated in Figure 3 and Figure 4.

The chemical additives could alter the hydrate phase equilibrium if they change the water activity, act as hydrate guests, or join hydrate structures. Nevertheless, the nanomaterials remain in their solid phase suspended in the liquid solution. Moreover, the molecular size and chemical structure do not allow solid particles to occupy water cavities or contribute to hydrate structures. Moreover, their immiscibility with water and the low concentration used remain the water activity unchanged as it depends on the composition of the mixture and the experimental conditions. Therefore, the nanofluids had a negligible impact on phase boundaries; consequently, they did not shift the equilibrium curve.

### 3.3. Induction Time Measurement

The induction times of the CH_4_ and CO_2_ hydrate formations are presented in Table 2. It can be noted that apart from the 0.05 wt% and 0.1 wt% of silver, all the metallic-based fluids reduced the induction times of CH_4_ hydrates relative to the SDS and water samples. Table 2 exhibits that 0.01 wt% of the Cu had the shortest induction time among the studied samples. The shortest induction time for Ag and Cu samples was found at 0.01 wt%.

The induction time measurements for CO_2_ hydrates demonstrate that the hydrate formed after about 65 min for all samples. The only considerable impact has been observed for 0.01 wt% Cu among all the kinetic promoters. As explained in the procedure, after getting to 274.15 K, the temperature was held for 65 min, and then the stirrer was turned on. This holding time was sufficient for developing the hydrate nucleus of CO_2_ due to its high solubility in water. Therefore, massive hydrate growth was observed immediately after the stirrer was turned on, providing proper agitation in the cell. A relevant study has noted the minor effect of kinetic promoters on CO_2_ hydrates [28]. Meanwhile, a remarkable impact of surfactants, such as SDS, has been observed on hydrocarbon guested hydrate [36].

Furthermore, it is essential to note that the induction time reported for Cu at the concentration of 0.01 wt% was less than 65 min. This refers to the CH_4_ and CO_2_ hydrate formation being started before agitation using the stirrer. That was not noticed for 0.03 wt% SDS. For industrial applications, the ability to form hydrates in a straightforward quiescent system is advantageous. The stirring device has some technical issues even though it can increase the formation rate. For instance, the viscosity could be increased during the hydrate production due to the generation of a thick hydrate slurry. This result in a dramatic increase in the energy demand to operate a more powerful impeller to achieve the proper mixing. In addition, forming a certain amount of hydrate around the impeller stops the rotation. Hence, mechanical mixing does not work anymore to complete the hydration process. Furthermore, avoiding mechanical agitation would reduce the risk of gas leakage, sparks issues, and static electricity, particularly when flammable and hazardous gases are used under pressure [32]. Not to mention that installing a stirrer could increase equipment capital and maintenance costs. Therefore, having metallic particles could help to act as a stirrer due to the Brownian motion [37].

This impact of nanoparticles on the induction time could be attributed to the changes in the intermolecular forces between water molecules near the surface of solid particles. These water molecules are attached to the surface of particles either by hydrogen bonds or adsorbed via Van Der Walls forces [38]. Therefore, the adsorbed water molecules aligned on the surface of the nanoparticles. These aligned water molecules with less freedom of movement could act as the building block of hydrate crystals, i.e., hydrate nucleus, and consequently reduce the induction time. However, increasing the concentration or size of nanoparticles could lead to more aggregation, reducing water activity and inhibiting hydrate formation [39]. Hence, certain concentrations like 0.01 wt% that were observed in this work could show the best promotion effect.

### 3.4. Initial Rate of the Gas Consumption

**Figure 5:**
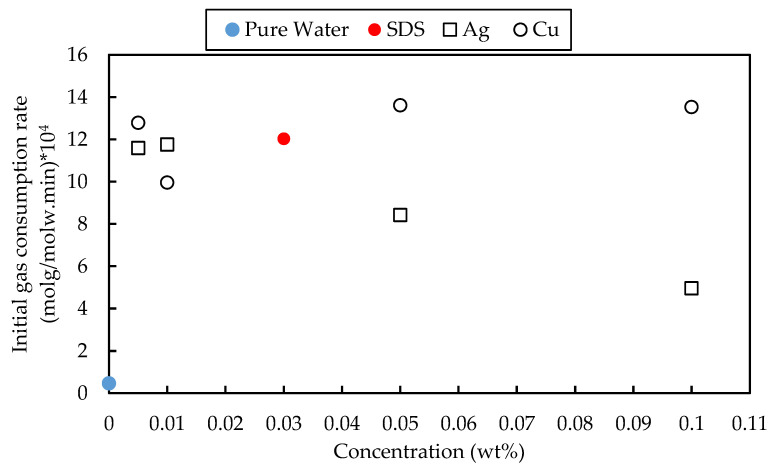
The initial CH_4_ consumption rate for the pure water, 0.03 wt% SDS, and metal-based fluid (0.005–0.1 wt% metallic particle + 0.03 wt% SDS).

The initial rate of CH_4_ gas consumption is seen in Figure 5. It is worth highlighting that all nanofluid samples contained 0.03 wt% SDS, and thus all samples improved the hydrate formation rate compared to water. In comparison to SDS, the solid particles did not show a significant promotion effect as they mainly diminished the CH_4_ hydrate formation rate. The maximum enhancement rate of 13.3% was observed at 0.05 wt% of Cu. In contrast, Ag deteriorates the performance of SDS as it reduces the initial gas consumption rate. The non-monotonic trend of initial formation rate vs. concentration is common. Cu and Ag belong to group 1B of transient metals with the same number of electrons in the valence band. However, Cu molecules with smaller sizes have higher charge densities. In addition, Cu particles are normally covered by a layer of copper oxide due to their high reactivity with oxygen, which enhances the stability of the particles in the aqueous suspension [40]. Both factors enhance nanofluid’s stability, resulting in a higher thermal conductivity and, subsequently, better evacuation of the heat generated during hydrate formation. This could increase the hydrate growth rate in the presence of Cu particles.

It is worth mentioning that the SDS effect on the initial gas consumption rate is more dominant, and therefore, the effect of particles as the nucleation sites is less important. However, in the absence of SDS, the nature and strength of intermolecular forces between solid particles and water molecules play a more important role [7]. The key part of the surfactant structure is the hydrophobic tail that could have boosted the dissolution of the hydrocarbon gases in the aqueous phase and resulted in hydrates’ promotion. On the other hand, SDS’s superiority over metallic-based fluids lies in its impact on the morphology of the produced CH_4_ hydrate. The presence of SDS pushed the produced hydrate towards the reactor wall, increasing the interface area between the phases and, consequently, enhancing the kinetics of the hydrate production. According to Siangsai et al., a thin layer of water associated with dissolved SDS molecules is adsorbed on the metal surface. It causes a capillary effect pushing more water molecules and hydrates crystals toward the reactor wall [41]. Contradictory, Ag or Cu nanoparticles act as the nucleation sites where hydrate could form around them and disturb the fast crystal growth along the wall due to mass transfer resistance.

**Figure 6:**
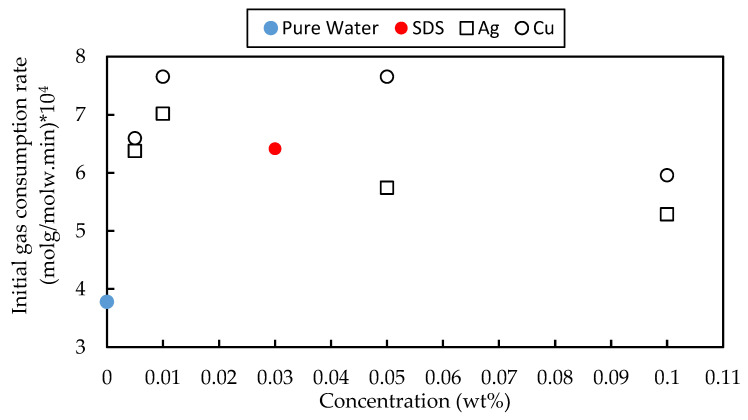
The initial CO_2_ consumption rate for the pure water, 0.03 wt% SDS, and metal-based fluid (0.005–0.1 wt% metallic particle + 0.03 wt% SDS).

Similar to CH_4_ results, all samples, including 0.03 wt% SDS or metal-based fluids, increased the initial rate of CO_2_ hydrate formation compared to water, as shown in Figure 6. Compared to SDS, copper enhanced the initial CO_2_ consumption rate at 0.005, 0.01, and 0.05 wt%, while silver is effective at 0.01 wt%. The maximum enhancement rate was observed for metal-based particles for 0.05 wt% Cu as it increased the initial rate by 102.6% and 19.4% relative to water and 0.03 wt% SDS, respectively. It is worth noting that, in the case of CO_2_ hydrates, the surface area of the hydrate chunk does not change significantly during hydrate formation as the morphology is nearly identical for pure water, SDS, or metal-based fluid. Hence, unlike methane hydrate, the morphology does not affect CO_2_ hydrate formation.

### 3.5. Half-Completion Time t_50_ and Semi-Completion Time t_95_ Measurements

The half-completion time reduction is vital for industrial applications as the slow nucleation and initial growth rate of hydrate remain the main challenge. The half-completion time of CH_4_ hydrates for the water sample is 656.1 min, which is reduced significantly by using chemical additives. Table 3 demonstrates that 0.01 wt% Ag showed the shortest *t*_50_ for CH_4_ hydrates compared to the other samples. Another parameter was studied, namely, the semi-completion time *t*_95_. *t*_95_ denotes the time required to reach 95% of the final gas uptake and storage capacity. The results showed that the water sample needs 1937.6 min to achieve 95% of CH_4_ hydrates formation. The shortest *t*_95_ observed for metal-based fluid is recorded for 0.01 wt% Ag, as seen in Table 3. At the beginning of the hydrate formation process, the crystal grew rapidly and consumed more gas, which caused the pressure to drop significantly. As a result, the sub-cooling degree was decreased, and the hydrate formation took a long time to complete. However, for an industrial scale, where a continuous process is more applicable, achieving the maximum conversion necessitates a longer retention time and reactor size, resulting in additional CAPEX. Therefore, attaining 95% of the hydrate formation in the reactor may be more economical than a 100% conversion. Consequently, shorter *t*_50_ and *t*_95_ are more favorable for reactor design purposes.

**Figure 7:**
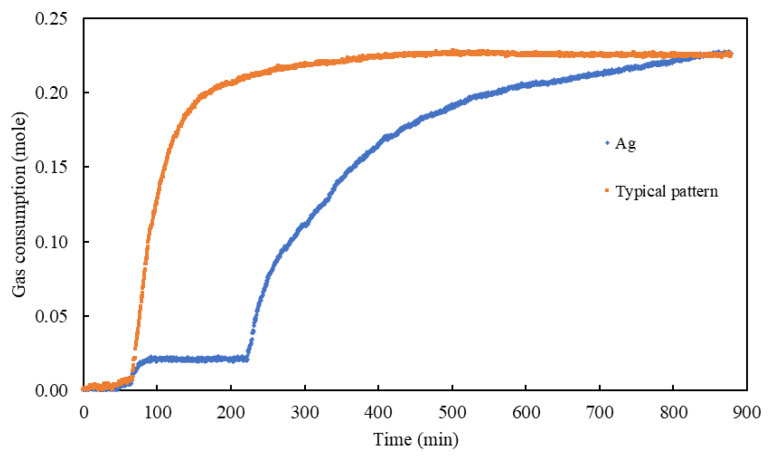
Comparison of CO_2_ gas consumption profile in the presence of 0.05 wt% Ag + 0.03 wt% SDS and typical hydrate formation pattern.

For CO_2_ hydrates, all additives reduced *t*_50_ relative to water except for Ag samples in the concentration range 0.01–0.1 wt%. The observed gas consumption profile of the Ag sample indicated that the CO_2_ hydrates formed in two stages. The pattern plotted in Figure 7 demonstrates that rapid hydrates formation occurs at the liquid/gas interface, which hinders further mass transfer.

Relative to SDS samples, only 0.005 wt% Ag decreased slightly *t*_50_ for one minute, possibly due to experimental error. The trend is different for *t*_95_ as it shows that 0.005 wt% of Cu has the lowest *t*_95_ value. It can be concluded that the metallic particles do not have a promotion effect. They either do not change *t*_50_ and *t*_95_ or slow the hydrate growth.

### 3.6. Gas Uptake and Storage Capacity

The gas uptake was calculated according to Equation (3). Once the nucleation stage is over, the hydrates start to form rapidly, and the pressure drops significantly due to the fast gas consumption. The pressure keeps dropping till the system reaches the equilibrium state. Due to the nature of isochoric experiments, the final gas consumption could be limited by the thermodynamic constrain of the system as the pressure reached about 1.38 MPa and 2.90 MPa for CO_2_ and CH_4_, respectively, which is the equilibrium pressure at 274.15 K. The final amount of gas consumption of all experiments reached almost the same value with a minor difference

Since the final gas consumption for all chemical additives reached to close point, the range of final gas consumption, gas uptake, and storage capacity of CO_2_ and CH_4_ hydrates were listed in Table 4.

The maximum theoretical SC of structure sI is ≈170 *v*/*v*. Hence, the maximum storage ratio achieved in this work is 95.2% and 91.0% for CH_4_ and CO_2_, respectively. There is no appreciable difference between the blank sample and the metal-based fluids for CO_2_ gas due to its high solubility in water. However, the difference is more pronounced for CH_4_ hydrate for the final gas uptake and storage capacity. It is also worth noting that the storage capacity of CH_4_ is higher than CO_2_. This is attributed to the cage occupancy in the cavities. It is worth highlighting that CH_4_ and CO_2_ can reside in small and large cavities of structure I. However, the larger size of CO_2_ molecules limits their occupancy in the small cavities, while CH_4_ can occupy both readily [9].

### 3.7. Modelling of Apparent Rate Constant

The apparent rate constant *K_app_* of pure CH_4_ and pure CO_2_ hydrate formation was calculated for samples containing solid particles Ag or Cu along with pure water and 0.03 wt% SDS samples (Table 5). The experimental data of *K_app_* for each sample was calculated using Equation (13) at each point. The average value was reported, and a constant initial consumption rate was assumed for prediction. The average apparent rate constant for methane hydrate formed in pure water is 0.17 mmol·min^−1^·MPa^−1^. The highest average apparent rate constant is observed for CH_4_ hydrate formation in the presence of the aqueous solution of Cu with a concentration of 0.05 and 0.1 wt%, which is about 5.89 mmol·min^−1^·MPa^−1^. The average apparent rate constant for carbon dioxide hydrate formed in pure water is about 3.17 mmol·min^−1^·MPa^−1^. The highest average apparent rate constant is observed for CO_2_ hydrate formation in the presence of the aqueous solution of Cu with a concentration of 0.05 wt%, which is 4.654 mmol·min^−1^·MPa^−1^.

The *K_app_* could be used later to find the reaction rate within the tested concentration range at 274.15 K. The correlation between the rate constant, *K_app_*, and the concentration, *C*, was found to be more practical. Using empirical formulas has the advantage of having a fast and reliable mathematical formula to interpolate between experimental points. There are, however, some limitations (such as extrapolations). The empirical equation is commonly used to calculate the rate constant without referring to the concentration. The correlation between the apparent rate constant, *K_app_*, and the concentration, *C*, was identified. The applied correlation between the apparent gas consumption rate constant as a function of concentration is presented as Equation (15):

(15) $$K_{app}=k_{0}+k_{1}C+k_{2}C^{2}$$

where *K_app_* is the apparent rate constant, *C* is the concentration wt% of nanomaterial, and *k*_0_, *k*_1_, and *k*_2_, are the regression coefficients.

The models are then used to estimate the new *K_app_* at each concentration and compare it with the calculated value by the Englezos model. The maximum reported error is 7.9%. For further validation of the proposed model, the concentration of metal particles is assumed to be zero, which corresponds to the 0.03 wt% SDS and the *K_app_* value, (*K_app_* = *k*_0_), compared with the calculated *K_app_* for the SDS experiments. The maximum error was found to be 14.14, which is very reasonable for kinetic models. The MAPE for the applied models is listed in Table 6.

As the nanomaterials have high thermal conductivity and the experiments are conducted in the stirred reactor, the heat transfer does not play the same important role as the mass transfer. Hence, the mass transfer-based model is confirmed as an appropriate predictive tool. As the Englezos model uses the fugacity difference as the driving force, the model combined the mass transfer coefficient around the particle *k_d_*, where *f_g_* − *f_s_* is the driving force, with *k_r_*, intrinsic rate constant where *f_s_* − *f_eq_* is the driving force. If the experiments are carried out under conditions such that heat and mass transfer resistances around the particle are eliminated, then *kd* >> *kr* and *K*^∗^ ≈ *k_r_*. From the study of Englezos et al., it was found that; in their apparatus, heat and mass transfer effects become negligible at stirring rates of 400 rpm or greater [33]. For the current study, it is expected that heat and mass transfer resistances will also be minor at a stirring rate of 600 rpm in the existing apparatus.

## 4. Conclusions

In this study, the impact of Cu and Ag particles on CH_4_ and CO_2_ hydrate formation was investigated. The metal-based fluids were prepared by suspending 0.005–0.1 wt% of solid particles in aqueous solutions of 0.03 wt% SDS. The thermodynamic results showed that metal-based fluids at a concentration of 0.1 wt% do not alter the hydrate equilibrium conditions. As this study compared the kinetic effect of silver and copper on both CH_4_ and CO_2_ hydrates, it can be concluded that Cu and Ag could promote or inhibit hydrate formation depending on the type of gas and particle concentration. Cu showed a significant promotional effect on CH_4_. Low concentrations favor hydrate promotion while higher concentrations predominantly inhibit the formation of hydrate. On the other hand, both Cu and Ag do not promote CO_2_ formation. They slow the kinetics of CO_2_ hydrate or do not show a significant impact. The higher charge density and the reactive surface of copper give merit over Ag for hydrate promotion. Copper could be a potential promoter due to its availability and low cost.

An empirical correlation was developed to predict the rate constant as a function of concentration. The rate constant was successfully predicted the rate constant with an acceptable error.

## Figures and Tables

**Table 1:** The properties of chemicals used in this study.

Chemical	Abbreviation	Diameter/nm	Purity	Supplier
Silver	Ag	30–50	>95%	Research Nanomaterials Inc. US
Copper	Cu	40	>95%	
Sodium dodecyl sulfate	SDS	NA	99%	Merck
Methane	CH_4_	NA	99.95%	Air Product Sdn. Bhd
Carbon dioxide	CO_2_	NA	99.95%	

**Table 2:** The induction time of CH_4_ and CO_2_ hydrates for blank, 0.03 wt% SDS, and solid-based fluids.

Aqueous Phase	Solid Phase	CH_4_			CO_2_		
		Mean Induction Time (min)	STD	Number of Runs	Mean Induction Time (min)	STD	Number of Runs
Pure Water	---	74.2	2.37	4	68.2	2.83	4
0.03 wt% SDS	---	73.9	5.96	5	65	0.05	3
0.03 wt% SDS	0.005 wt% Ag	66	0.26	3	66	0.82	3
	0.01 wt% Ag	62.7	2.76	4	66.2	0.56	3
	0.05 wt% Ag	77.2	3.55	4	65.3	0.47	3
	0.1 wt% Ag	76.1	4.69	5	64.6	0.83	4
	0.005 wt% Cu	66.1	0.65	3	66	0.12	3
	0.01 wt% Cu	56.8	5.13	4	57.8	6.72	3
	0.05 wt% Cu	65	3.49	4	65	0.47	3
	0.1 wt% Cu	65	2.48	4	65.1	0.41	3

**Table 3:** Mean half-completion time and mean semi-completion time of CO_2_ and CH_4_ hydrate with the standard deviation STD for pure water, 0.03 wt% SDS, and 0.005–0.1 wt% solid particles.

Aqueous Phase	Solid Phase	Mean *t*_50_	STD	Mean *t*_95_	STD	Number of Runs
		CO_2_				
Pure Water	---	117.0	3.74	518.0	24.65	4
0.03 wt% SDS	---	100.3	3.14	246.7	21.01	3
0.03 wt% SDS	0.005 wt% Ag	99.0	4.23	250.0	16.02	3
	0.010 wt% Ag	137.6	8.82	340.9	26.12	3
	0.050 wt% Ag	207.7	38.92	575.4	129.29	5
	0.100 wt% Ag	137.3	35.00	378.9	58.67	4
	0.005 wt% Cu	103.0	8.93	240.0	3.50	3
	0.010 wt% Cu	102.0	6.44	266.5	61.50	3
	0.050 wt% Cu	109.5	1.50	308.5	8.50	3
	0.100 wt% Cu	108.8	0.01	285.5	13.52	3
		CH_4_				
Pure Water	---	656.1	89.26	1937.6	110.19	4
0.03 wt% SDS	---	107.0	10.48	149.4	30.57	5
0.03 wt% SDS	0.005 wt% Ag	92.7	3.55	135.7	3.32	3
	0.010 wt% Ag	89.4	20.29	128.4	17.50	4
	0.050 wt% Ag	150.3	29.85	195.7	11.42	4
	0.100 wt% Ag	152.1	33.71	200.3	11.25	5
	0.005 wt% Cu	91.0	1.91	137.5	6.12	3
	0.010 wt% Cu	98.6	3.73	150.6	10.33	4
	0.050 wt% Cu	95.2	6.63	136.0	9.02	4
	0.100 wt% Cu	114.2	8.87	157.2	8.55	4

**Table 4:** Gas consumption, gas uptake, and the storage capacity of CH_4_ and CO_2_.

Sample	Hydrate Former	Gas Consumption (mol)	Gas Uptake (mol_g_/mol_w_)	Storage Capacity *v*/*v*
Pure water	CH_4_	0.2087	0.0380	113.9
	CO_2_	0.2240	0.0409	151.8
With kinetic promoters	CH_4_	0.3320–0.3420	0.0606–0.0625	159–163.7
	CO_2_	0.2240–0.2300	0.0409–0.0420	151.8–155.9

**Table 5:** The apparent rate constant of CH_4_ and CO_2_ hydrates calculated using the Englozes model.

Aqueous Phase	Solid Phase	Apparent Rate Constant *K_app_* (mol/min MPa)	
		CH_4_	CO_2_
Pure water	----	0.00017	0.00317
0.03 wt% SDS	----	0.00547	0.00489
0.03 wt% SDS	0.005 wt% Ag	0.00460	0.00390
	0.010 wt% Ag	0.00554	0.00435
	0.050 wt% Ag	0.00400	0.00376
	0.100 wt% Ag	0.00192	0.00341
	0.005 wt% Cu	0.00516	0.00465
	0.010 wt% Cu	0.00496	0.00551
	0.050 wt% Cu	0.00589	0.00565
	0.100 wt% Cu	0.00589	0.00426

**Table 6:** Regression coefficients of the correlation between apparent rate and concentration and the resulting mean absolute percentage error (MAPE%).

Nanomaterial	Gas	Regression Coefficients			MAPE%	MAPE% (SDS)
		*k*_0_	*k*_1_	*k*_2_		
Ag	CH_4_	0.0051	−0.0079	−0.2416	7.90	6.75
	CO_2_	0.0042	−0.0061	−0.0147	5.18	14.14
Cu	CH_4_	0.0049	0.0291	−0.1878	2.50	10.41
	CO_2_	0.0048	0.0437	−0.4884	5.37	1.87

## Data Availability

The data presented in this study are available on request from the corresponding author.
